# Cancer-associated fibroblasts promote an immunosuppressive microenvironment through the induction and accumulation of protumoral macrophages

**DOI:** 10.18632/oncotarget.14374

**Published:** 2016-12-30

**Authors:** Hideyuki Takahashi, Koichi Sakakura, Takeshi Kudo, Minoru Toyoda, Kyoichi Kaira, Tetsunari Oyama, Kazuaki Chikamatsu

**Affiliations:** ^1^ Department of Otolaryngology-Head and Neck Surgery, Gunma University Graduate School of Medicine, Gunma, Japan; ^2^ Department of Oncology Clinical Development, Gunma University Graduate School of Medicine, Gunma, Japan; ^3^ Department of Pathology, Gunma University Graduate School of Medicine, Gunma, Japan

**Keywords:** cancer-associated fibroblast (CAF), tumor-associated macrophage (TAM), tumor microenvironment (TME), immunomodulation, immunosuppression

## Abstract

Stromal cells in the tumor microenvironment (TME) closely interact with tumor cells and affect tumor cell behavior in diverse manners. We herein investigated the mechanisms by which cancer-associated fibroblasts (CAFs) affect the functional polarization of tumor-associated macrophages (TAMs) in oral squamous cell carcinoma (OSCC) *in vitro* and in human cancer samples. The expression of CD68, CD14, CD163, CD200R, CD206, HLA-G, CD80, and CD86 was higher in CD14-positive cells co-cultured with the culture supernatants of CAFs established from OSCC specimens (CAF-educated cells) than in control cells. The gene expression level of *ARG1*, *IL10*, and *TGFB1* was increased in CAF-educated cells. CAF-educated cells suppressed T cell proliferation more strongly than control cells, and the neutralization of TGF-β IL-10, or arginase I significantly restored T cell proliferation. We then investigated the relationship between the infiltration of CAFs and TAMs using tissue samples obtained from patients with OSCC. The infiltration of CAFs was associated with the numbers of CD68-positive and CD163-positive macrophages. It also correlated with lymphatic invasion, vascular invasion, lymph node involvement, and the TNM stage. The infiltration of CAFs was identified as an independent prognostic factor in OSCC. Our results indicate that CAFs play important roles in shaping the tumor immunosuppressive microenvironment in OSCC by inducing the protumoral phenotype of TAMs. Therapeutic strategies to reverse CAF-mediated immunosuppression need to be considered.

## INTRODUCTION

Stromal cells in the tumor microenvironment (TME) closely interact with tumor cells and affect tumor cell behavior in diverse manners; for example, they promote tumor growth, invasion, and metastasis, in addition to treatment resistance [1, 2]. However, the relationship between the TME and the immune system is more complex and has not yet been elucidated. Tumor tissue is infiltrated by a number of immune cells that show functional plasticity and may adopt antitumor or protumor activity. Emerging evidence suggests that stromal cells, including fibroblasts, endothelial cells, and mesenchymal stem cells, play pivotal roles in shaping the tumor immune environment [3, 4]. Fibroblasts have been identified as one of the most active cell types of the tumor stroma [1, 2, 5]. In the TME, fibroblasts transdifferentiate into activated phenotype myofibroblasts through transforming growth factor beta (TGF-β) and interleukin (IL)-1 beta signaling [6, 7], and are known as cancer-associated fibroblasts (CAFs). We recently demonstrated that CAFs in squamous cell carcinoma of the head and neck directly and indirectly modulate effector T cell function during antitumor immune responses [8]. CAFs exhibit greater suppressor activity on T cell proliferation through co-regulatory molecules and immunosuppressive cytokines than normal fibroblasts. Moreover, CAFs preferentially induce T cell apoptosis and regulatory T cells. Similar findings have also been demonstrated in CAFs obtained from melanoma and non-small cell lung cancer [9, 10].

In order to investigate crosstalk between CAFs and immune cells in the TME in more detail, we focused on macrophages in the present study. Among the inflammatory cells recruited to the tumor site, macrophages are the most abundant cell type and are called tumor-associated macrophages (TAMs) [11, 12]. TAMs comprise two functionally paradoxical phenotypes: M1 and M2 macrophages. M1 macrophages are induced in response to interferon-γ and lipopolysaccharide, and exert anti-tumor responses through the production of pro-inflammatory cytokines, nitric oxide, and reactive oxygen intermediates. In contrast, M2 macrophages are induced in response to various signals such as IL-4, IL-10, and IL-13, and perform immunosuppressive functions, stimulate angiogenesis, and enhance tumor cell invasion [11, 12]. Therefore, a number of microenvironmental factors released from CAFs as well as tumor cells in the TME may be the major determinants of TAM polarization and functions during the differentiation of circulating monocytes to TAMs. Our previous findings indicated that CAFs showed increased expression levels of *IL6*, *CXCL8*, and *TGF1* [8]. Inflammatory cytokines, such as IL-6 and CXCL8, are known to support M2 macrophage polarization [13–15], whereas TGF-β recruits and retains macrophages at the tumor site and enables effective tumor evasion of the host immune system [16, 17]. Thus, CAFs may have profound effects on the polarization and recruitment of TAMs. The aim of the present study was to investigate the relationship between CAFs and TAMs in oral squamous cell carcinoma (OSCC) *in vitro* and in human cancer samples. CAFs established from the resected tumor tissues of patients with OSCC preferentially induced the protumoral and immunosuppressive phenotype of macrophages from circulating monocytes. Moreover, the infiltration of CAFs in tumor tissue correlated with the number of not only CD68+ macrophages, but also CD163+ macrophages, indicating that CAFs skew toward M2 macrophages in the TME. The results of the present study have provided novel insights into the role of CAFs in the tumor immunosuppressive microenvironment. Therapeutic strategies to reverse the CAFs-mediated immunosuppressive microenvironment need to be considered in order to increase the effectiveness of immunotherapies.

## RESULTS

### Establishment of CAFs and their characteristics

**Figure 1:**
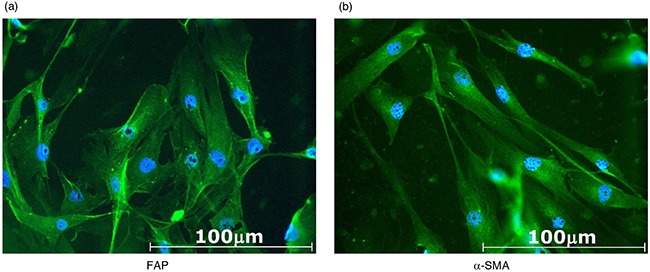
Representative photomicrographs of CAF3 established from resected tumor samples of patients with OSCC CAFs were cultured in a chamber slide for 48 hours, then stained for **a.** fibroblast activation protein (FAP) or **b.** α-smooth muscle actin (α-SMA). CAFs were positive for FAP and α-SMA.

Two cell lines of CAFs were generated from the resected tumor samples of patients with OSCC, and named CAF3 and CAF4. These cells grew in primary cultures in an adherent manner and possessed a fibroblast-like morphology. Cells were analyzed by flow cytometry to confirm that they were activated fibroblasts and not contaminated by leukocytes, endothelial cells, or tumor cells. CAFs were negative for CD11b, CD34, and CD45, and positive for CD90, fibroblast activation protein (FAP), and α-smooth muscle actin (α-SMA), as previously described [8]. CAFs were also evaluated by immunocytochemistry, and were positive for FAP and α-SMA (Figure 1). These results confirmed the identity of the cultures as CAFs for further assays.

### CAF-educated cells phenotypically resembled protumoral macrophages

**Figure 2:**
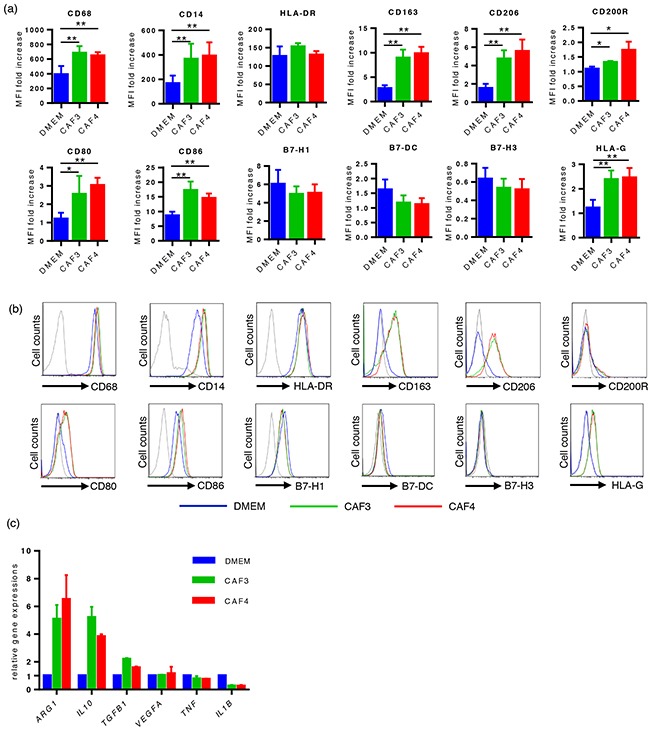
The culture supernatants of CAFs induced the protumoral phenotype of macrophages CD14-positive PBMCs prepared from healthy donors were cultured with culture supernatants from CAFs (CAF-educated cells) or DMEM (control cells) for 48 hours, then analyzed using flow cytometry or real-time quantitative RT-PCR. **a.** The expression levels of CD68, CD14, CD163, CD206, CD200R, HLA-G, CD80, and CD86 were significantly higher in CAF-educated cells than in control cells. Data are presented as relative fold changes to control IgG in mean fluorescent intensity (MFI). **b.** Representative histograms of each molecule are shown. **c.** The gene expression levels of ARG1, IL10, and TGFB1 were higher in CAF-educated cells than in control cells. *P < 0.05, **P < 0.01.

We developed an *in vitro* model using CD14-positive cells prepared from healthy donors and culture supernatants from CAFs to investigate the influence of CAFs on the polarization of macrophages. After 48 hours of cultivation, the expression of myeloid cell markers, including CD68, CD14, CD163, CD200R, and CD206, was stronger in CAF-educated cells than in control cells, whereas that of human leukocyte antigen (HLA)-DR was similar between the two groups (Figure 2a–2b). The expression of HLA-G, CD80, and CD86 was also stronger in CAF-educated cells than in control cells, whereas that of B7H1/PD-L1, B7DC/PD-L2, and B7H3 was similar among the two groups (Figure 2a–2b). The gene expression levels of the enzyme and cytokines were also analyzed by real-time quantitative RT-PCR after 48 hours of cultivation. The gene expression levels of *ARG1*, *IL10*, and *TGFB1* were higher in CAF-educated cells than in control cells, whereas that of *IL1B* was lower in CAF-educated cells than in control cells (Figure 2c). These results indicate that the culture supernatants of CAFs induced the protumoral phenotype of macrophages.

### CAF-educated cells were potent suppressors of autologous T cells

**Figure 3:**
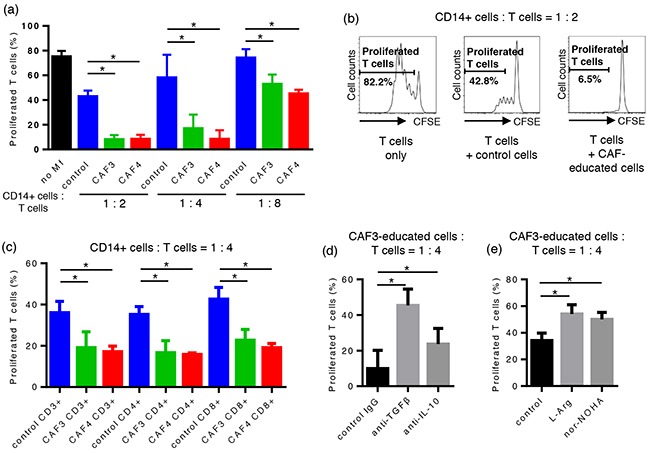
CAF-educated cells were potent suppressors of autologous T cells CAF-educated cells or control cells were co-cultured with carboxyfluorescein succinimidyl ester (CFSE)-labeled autologous CD14-negative cells for 96 hours with an anti-CD3/anti-CD28 stimulus. **a.** The proliferation of T cells was suppressed by CAF-educated cells more strongly than control cells in a dose-dependent manner. **b.** Representative histograms of the proliferation of T cells. **c.** The proliferation of both CD4+ T cells and CD8+ T cells was equally suppressed by CAF-educated cells compared with control cells. **d.** Neutralizing mAbs were added during the co-cultivation of CAF-educated cells and CFSE-labeled cells. The addition of anti-TGF-β mAb or anti-IL-10 mAb significantly restored the proliferation of T cells. **e.** The addition of the arginase I inhibitor Noha or L-arginine during co-cultivation significantly restored T cell proliferation. *P < 0.05.

In order to evaluate the effects of CAF-educated cells on T cell proliferation, CAF-educated cells or control cells were co-cultured with carboxyfluorescein succinimidyl ester (CFSE)-labeled autologous CD14-negative cells for 96 hours with an anti-CD3/anti-CD28 stimulus. At a 1:2 CAF-educated cells:CFSE-labeled cell ratio, the proliferation of T cells was suppressed by CAF-educated cells more strongly than by control cells: 7.9 ± 6.8% for CAF3-educated cells, 8.2 ± 5.7% for CAF4-educated cells, and 42.8 ± 9.8% for control cells (median ± interquartile range). The proliferation of T cells was significantly suppressed in a dose-dependent manner (Figure 3a–3b). The proliferation of both CD4+ T cells and CD8+ T cells was equally suppressed by CAF-educated cells compared with control cells (Figure 3c).

### CAF-educated cells suppressed T cell proliferation through the production of TGF-β, IL-10, and arginase I

In order to assess the suppressive mechanisms of CAF-educated cells on T cells, neutralizing mAbs, the arginase I inhibitor Noha, or L-arginine were added during the co-cultivation of CAF-educated cells and CFSE-labeled cells. The addition of anti-TGF-β mAb, anti-IL-10 mAb, Noha, and L-arginine significantly restored T cell proliferation, indicating that the suppressive activity on T cells mediated by CAF-educated cells is in part due to the production of TGF-β, IL-10, and arginase I (Figure 3d–3e).

### Patient characteristics

The clinicopathological characteristics of 73 patients are summarized in Table 1. Postoperative adjuvant chemotherapy with the oral administration of S-1 (Taiho Pharmaceutical Co., Ltd., Tokyo, Japan), tegafur, or docetaxel was given to 9, 10, and 3 patients, respectively. The median follow-up duration was 915 days (range, 85-3452 days).

**Table 1:** Characteristics of 73 patients

	No. (%)
Age, years	
Mean	69
Range	36-92
Gender	
Male	46 (63)
Differentiation	
Well/moderate	63 (86)
Poorly	10 (14)
Lymphatic invasion	34 (47)
Vessel invasion	24 (33)
Lymph node involvement	25 (34)
T factor	
T1-2	63 (86)
T3-4	10 (14)
TNM stage	
I-II	47 (64)
III-IV	26 (36)

### Expression of α-SMA, CD68, and CD163 in OSCC specimens

**Figure 4:**
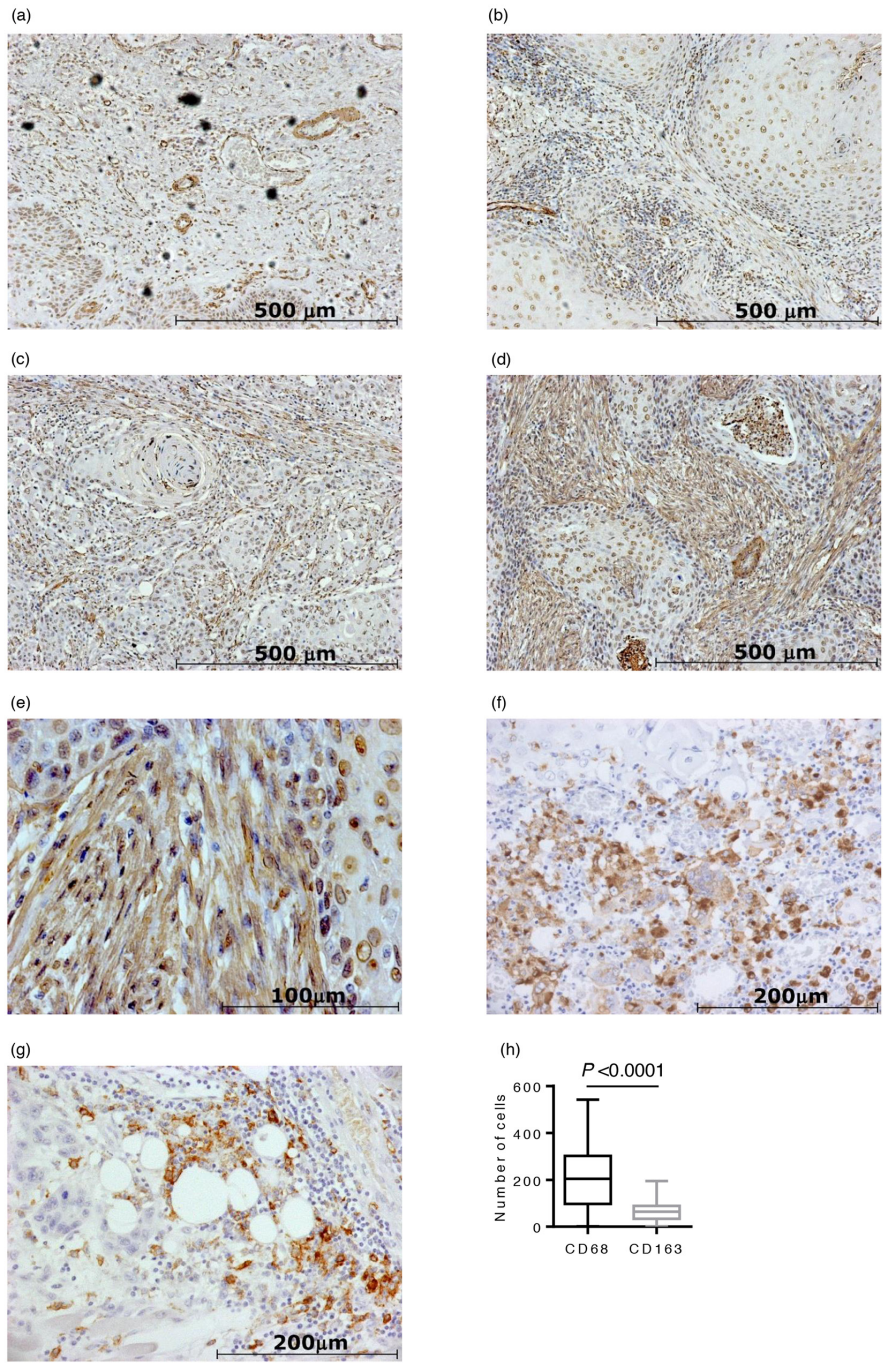
Immunohistochemical staining of 73 oral squamous cell carcinoma (OSCC) surgical specimens Representative photomicrographs of α-SMA staining are shown in (a)-(e): **a.** CAFs grade 0 case (×100 magnification). Blood vessel walls were used as an internal positive control for α-SMA; **b.** CAFs grade 1 case (×100 magnification); **c.** CAFs grade 2 case (×100 magnification); **d.** CAFs grade 3 case (×100 magnification); **e.** CAFs grade 3 case (×400 magnification). **f.** A representative photomicrograph of CD68 staining (×200 magnification). **g.** A representative photomicrograph of CD163 staining (×200 magnification). **h.** The number of CD68-positive macrophages was significantly higher than that of CD163-positive macrophages (P < 0.0001).

Representative immunohistochemistry (IHC) staining of α-SMA, CD68, and CD163 is shown in Figure 4. CAFs were identified by a spindle-shaped structure with an elongated nucleus and positive α-SMA staining (Figure 4e). Blood vessel walls were used as an internal positive control for α-SMA (Figure 4a). Eleven OSCC specimens (15%) had Grade 0 CAFs, 17 (23%) had Grade 1 CAFs, 26 (36%) had Grade 2 CAFs, and 19 (26%) had Grade 3 CAFs. The median number and interquartile range of CD68-positive and CD163-positive macrophages were 204 ± 200 and 64 ± 55, respectively (Figure 4h). The number of CD68-positive macrophages was significantly higher than that of CD163-positive macrophages (*P* < 0.0001).

### Relationships between the infiltration of CAFs, number of TAMs, and clinicopathological features

The results of a univariate analysis on the relationships between the CAFs grades, number of TAMs, and clinicopathological features of 73 patients with OSCC are shown in Table 2 and Table 3. The high grade of CAFs significantly correlated with the number of TAMs. The high grade of CAFs correlated with lymphatic invasion (*P* = 0.02), vascular invasion (*P* = 0.02), lymph node involvement (*P* = 0.04), and the tumor-node-metastasis (TNM) stage (*P* = 0.03). The high number of CD68-positive macrophages correlated with vascular invasion (*P* < 0.01), lymph node involvement (*P* = 0.04), the TNM stage (*P* = 0.02), and the Ki-67 labeling index (*P* = 0.01), while that of CD163-positive macrophages also correlated with vascular invasion (*P* < 0.01) and the Ki-67 labeling index (*P* < 0.01).

**Table 2:** Relationship between CAFs grades, the number of CD68-positive and CD163-positive macrophages, and clinicopathological parameters in OSCC patients

Variable	CAFs grades					CD68		CD163	
	0	1	2	3	*P*-value	Median (IQR)	*P*-value	Median (IQR)	*P*-value
Age (years)									
>=71	5	8	12	8	0.99	204 (196)	0.31	68 (75)	0.20
<71	6	9	14	11		200 (212)		59 (54)	
Gender									
Male	6	6	19	12	0.29	222 (221)	0.56	61 (55)	0.15
Female	5	8	7	7		173 (178)		77 (69)	
Differentiation									
Well/moderate	10	15	21	17	0.78	204 (210)	0.72	62 (54)	0.12
Poorly	1	2	5	2		201 (180)		93 (73)	
Lymphatic invasion									
Negative	10	8	15	6	**0.02**	153 (211)	0.09	58 (68)	0.17
Positive	1	9	11	13		236 (181)		68 (62)	
Vascular invasion									
Negative	11	13	16	9	**0.02**	153 (202)	**<0.01**	58 (55)	**<0.01**
Positive	0	4	10	10		251 (146)		83 (86)	
Lymph node involvement									
Negative	10	11	19	8	**0.04**	151 (191)	**0.04**	58 (61)	0.08
Positive	1	6	7	11		260 (159)		72 (63)	
T factor									
T1-2	11	17	20	15	0.06	185 (194)	0.21	65 (56)	0.66
T3-4	0	0	6	4		236 (139)		41 (68)	
TNM stage									
I-II	10	13	16	8	**0.03**	140 (177)	**0.02**	65 (65)	0.60
III-IV	1	4	10	11		262 (152)		62 (58)	
Ki-67									
<26	10	8	13	12	0.08	153 (187)	**0.01**	42 (52)	**<0.01**
>=26	1	9	13	7		236 (196)		86 (60)	
p53									
Negative	7	7	12	10	0.70	187 (206)	0.95	61 (68)	0..83
Positive	4	9	14	8		222 (199)		65 (54)	

Abbreviations: CAF, cancer-associated fibroblast; OSCC, oral squamous cell carcinoma; IQR, interquartile range.

**Table 3:** Relationship between CAFs grades and the number of macrophages

CAFs grades	CD68		CD163	
	Median (IQR)	*P*-value	Median (IQR)	*P*-value
0	93 (107)	**<0.01**	11 (28)	**<0.01**
1	197 (207)		74 (50)	
2	238 (243)		68 (88)	
3	227 (129)		65 (45)	

### Survival analysis in patients with OSCC

**Figure 5:**
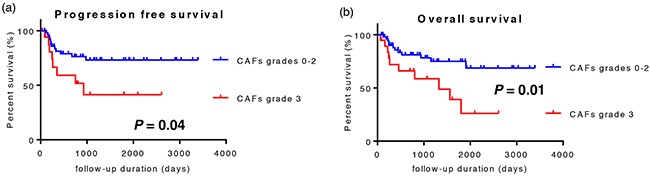
Kaplan-Meier survival analysis of 73 patients with OSCC Patients were divided into two groups: CAFs grades 0-2 cases and CAFs grade 3 cases. **a.** CAFs grade 3 cases showed significantly shorter progression-free survival. **b.** CAFs grade 3 cases showed significantly shorter overall survival.

Univariate/multivariate survival analyses on progression-free survival (PFS) and overall survival (OS) were performed using Cox's proportional hazards regression model in order to evaluate the prognostic value of CAFs grades, number of TAMs, and clinicopathological features. Receiver operating characteristic (ROC) curves were separately plotted to obtain the optimum cut-off point for the number of macrophages and the Ki-67 labeling index. The results of the survival analysis of PFS are shown in Table 4. Lymphatic invasion (*P* = 0.02), lymph node involvement (*P* < 0.01), the Ki-67 labeling index (*P* = 0.02), CAFs grades (*P* = 0.04), and the number of CD68-positive macrophages (*P* = 0.02) significantly influenced PFS in the univariate analysis, and CAFs grades were identified as an independent prognostic factor (*P* = 0.04). The results of the survival analysis of OS are shown in Table 5. Patient age (*P* = 0.05), the differentiation of tumors (*P* = 0.04), lymphatic invasion (*P* < 0.01), vascular invasion (*P* < 0.01), lymph node involvement (*P* = 0.03), T factor (*P* = 0.04), the TNM stage (*P* < 0.01), the Ki-67 labeling index (*P* < 0.01), and CAFs grades (*P* = 0.02) significantly influenced OS in the univariate analysis. Moreover, CAFs grades were identified as an independent prognostic factor (*P* < 0.01). In accordance with these results, a Kaplan-Meier survival analysis was also performed on CAFs grades (Figure 5). PFS and OS were significantly shorter in CAFs grade 3 cases than in CAFs grades 0-2 cases.

**Table 4:** Univariate and multivariate survival analyses on PFS in OSCC patients

Variables		Reference	Univariate			Multivariate		
			HR	95% CI	*P*-value	HR	95% CI	*P*-value
Age (years)								
	<71	>=71	0.882	0.365-2.132	0.78			
Gender								
	Female	Male	0.826	0.3168-2.152	0.70			
Differentiation								
	Poorly	Well/moderate	2.334	0.844-6.454	0.10			
Lymphatic invasion								
	Positive	Negative	**2.938**	**1.183-7.297**	**0.02**	1.533	0.408-5.766	0.53
Vascular invasion								
	Positive	Negative	1.914	0.779-4.699	0.16			
Lymph node involvement								
	Positive	Negative	**3.260**	**1.336-7.956**	**< 0.01**	1.542	0.412-5.776	0.52
T factor								
	T3-4	T1-2	0.833	0.193-3.606	0.81			
TNM stage								
	III-IV	I-II	1.770	0.717-4.370	0.22			
Ki-67								
	>=26	<26	**2.973**	**1.205-7.333**	**0.02**	2.180	0.800-5.945	0.13
p53								
	Positive	Negative	1.052	0.4266-2.594	0.91			
CAFs grades								
	Grade 3	Grades 0-2	**2.532**	**1.033-6.207**	**0.04**	**2.626**	**1.001-6.888**	**0.04**
CD68								
	>=145	<145	**4.307**	**1.259-14.742**	**0.02**	2.382	0.633-8.961	0.20
CD163								
	>=80	<80	2.304	0.957-5.543	0.06	1.322	0.524-3.338	0.55

Abbreviations: PFS, progression-free survival; OSCC, oral squamous cell carcinoma; HR, hazard ratio; CI, confidence interval; CAF, cancer-associated fibroblast.

**Table 5:** Univariate and multivariate survival analyses on OS in OSCC patients

Variables		Reference	Univariate			Multivariate		
			HR	95% CI	*P*-value	HR	95% CI	*P*-value
Age (years)								
	<71	>=71	**0.425**	**0.181-0.999**	**0.05**	0.624	0.224-1.735	0.37
Gender								
	Female	Male	0.984	0.398-2.428	0.97			
Differentiation								
	Poorly	Well/moderate	**2.771**	**1.070-7.174**	**0.04**	1.548	0.491-4.879	0.46
Lymphatic invasion								
	Positive	Negative	**3.745**	**1.522-9.219**	**< 0.01**	1.857	0.421-8.197	0.41
Vascular invasion								
	Positive	Negative	**4.138**	**1.747-9.802**	**< 0.01**	2.933	0.831-10.356	0.10
Lymph node involvement								
	Positive	Negative	**2.700**	**1.128-6.461**	**0.03**	0.820	0.174-3.860	0.80
T factor								
	T3-4	T1-2	**2.963**	**1.060-8.288**	**0.04**	1.022	0.231-4.522	0.98
TNM stage								
	III-IV	I-II	**3.188**	**1.338-7.593**	**< 0.01**	1.195	0.166-8.597	0.86
Ki-67								
	>=26	<26	**3.241**	**1.348-7.788**	**< 0.01**	3.114	0.978-9.918	0.06
p53								
	Positive	Negative	1.563	0.646-3.780	0.32			
CAFs grades								
	Grade 3	Grades 0-2	**2.809**	**1.210-6.521**	**0.02**	**3.872**	**1.428-10.498**	**< 0.01**
CD68								
	>=145	<145	2.332	0.859-6.332	0.10			
CD163								
	>=80	<80	2.335	0.993-5.490	0.05	1.114	0.345-3.597	0.86

Abbreviations: OS, overall survival; OSCC, oral squamous cell carcinoma; HR, hazard ratio; CI, confidence interval; CAF, cancer-associated fibroblast.

## DISCUSSION

The immunosuppressive status of the TME represents a major barrier for various immunotherapeutic approaches as well as conventional therapies [18]. Tumor-mediated immune suppressive mechanisms, including the expression of inhibitory molecules, secretion of immunosuppressive cytokines, and induction of immunosuppressive cellular populations, have been extensively elucidated [19, 20]. Similarly, stromal cells display multiple immunosuppressive mechanisms to evade anti-tumor immunity [8–10]. In the present study, we elucidated the mechanisms by which CAFs induced the protumoral and immunosuppressive phenotype of TAMs in OSCC *in vitro* and in human cancer samples. CD14-positive cells co-cultured with CAF supernatants up-regulated the expression of CD68, CD163, CD200R, and CD206, suggesting that monocytes differentiate into the protumoral phenotype of macrophages [21, 22]. The expression of non-classical class I molecule HLA-G was also up-regulated in CAF-educated cells. Several studies have reported the role of HLA-G as a molecule involved in immune tolerance, and the expression of HLA-G has been reported on both tumor cells and myeloid cells in the tumor microenvironment [11, 23]. Moreover, the expression of CD14, CD80, and CD86 was up-regulated in CAF-educated cells. Martinez et al. reported an increase in CD14 mRNA levels in human monocyte-derived macrophages polarized toward an M2 phenotype rather than an M1 phenotype, supporting supernatants from CAFs preferentially inducing the M2 phenotype [24]. In addition, CAF-educated cells also showed significant increases in CD80 and CD86 expression levels. M1 macrophages express CD80 and CD86, and a subset of M2 macrophages express CD86 [25, 26]. CAF-educated cells may be a heterogeneous cell population containing not only M2, but also M1 macrophages, and their activation status may increase the expression of CD80 and CD86.

The expression of the B7 family members B7H1/PD-L1, B7DC/PD-L2, and B7-H3, which are known as immunomodulatory molecules, did not increase on the surface of CAF-educated cells. Lyford-Pike et al. previously demonstrated that the expression of PD-L1 in head and neck squamous cell carcinoma was localized to TAMs as well as tumor cells [27]. In the TME, the functional phenotype of TAMs is regulated by a number of factors produced from tumor cells and stromal cells. Our results revealed that only CAFs may not be able to contribute to the expression of B7 family members on TAMs, suggesting that the induction of co-regulatory molecules on TAMs is required to ensure cooperation with tumor cells and/or other stromal cells.

We then assessed whether CAF-educated cells have immunosuppressive functions. As expected, the expression of *ARG1*, *IL10*, and *TGFB1*, which are known as effector molecules for the identification of M2 macrophages [11, 12], was stronger in CAF-educated cells than in control cells. Furthermore, CAF-educated cells significantly suppressed T cell proliferation, and the neutralization of IL-10, TGF-β or arginase I restored the inhibitory effects of CAF-educated cells. These results indicate that CAF-educated cells function as potent suppressors of T cell responses. Thus, CAFs seem to play a key role in the establishment of the immunosuppressive microenvironment through the induction of the protumoral and immunosuppressive phenotype of macrophages. In order to verify these results in human cancer samples, we investigated the relationship between the infiltration of TAMs and CAFs using tissue samples obtained from patients with OSCC. According to previous findings, the numbers of all macrophages and M2 macrophages in OSCC specimens were evaluated by the expression of CD68 and CD163, respectively [28, 29]. As expected, the high grade of CAFs significantly correlated with the number of TAMs; on the other hand, the median numbers of both CD68-positive and CD163-positive macrophages were highest in specimens with Grade 2, but not Grade 3 CAFs, suggesting that CAFs contribute to the recruitment, proliferation, and M2 skewing of macrophages in the TME. However, an excessive imbalance in components in the TME, such as the strong infiltration of CAFs, may cause distributional and/or functional changes in other components including TAMs.

This study firstly indicated that the numbers of total and M2-migrated macrophages strongly correlated with vascular invasion in OSCC specimens. The findings of several experimental studies support this result: (1) a paracrine loop that consists of tumor-cell-synthesized colony-stimulating factor 1 (CSF-1) and macrophage-derived epidermal growth factor (EGF) promotes tumor cell migration and invasion [30]; (2) macrophage-derived osteonectin (SPARC) increases the tumor cell-extracellular matrix (ECM) interaction, resulting in the migration of tumor cells [31]; (3) elevated cathepsin protease activities in macrophages enhance tumor invasion [32]; and (4) TGF-β secreted from macrophages promotes the epithelial-mesenchymal transition (EMT) of the invading tumor cells [33]. Thus, TAMs in OSCC, particularly those with the M2 phenotype, may play important roles in tumor vascular invasion through multiple mechanisms, resulting in poor prognosis.

Similar to the findings of Kellermann [34], the high grade of CAFs correlated with lymph node involvement and an advanced TNM stage. Moreover, we revealed that the infiltration of CAFs correlated with vascular invasion and lymphatic invasion. CAFs have the capacity to directly facilitate tumor invasion through the production of proteases including matrix metalloproteases (MMP), which help digestion of ECM [35], and produce various pro-invasive molecules [36–39]. These findings indicate that CAFs may promote cancer invasion, resulting in poor prognosis in patients with OSCC.

In conclusion, we herein showed that CAFs play pivotal roles in shaping the tumor immunosuppressive microenvironment in OSCC, and therapeutic strategies to reverse the CAF-mediated immunosuppressive microenvironment need to be considered in order to increase the effectiveness of conventional therapies as well as immunotherapies.

## MATERIALS AND METHODS

### Establishment of CAFs

Tumor tissues were obtained from two newly diagnosed OSCC patients who underwent surgery at the Department of Otolaryngology-Head and Neck Surgery, Gunma University Hospital. Patients had received no anticancer drugs or radiotherapy before surgery. CAF cell lines were established from each tissue according to a previously described method [8], and named CAF3 and CAF4. The expression of several markers on CAFs was evaluated using flow cytometry, as described previously [8], and immunocytochemistry. CAFs used in the present study were from less than 10 passages. Culture supernatants were collected from semi-confluent cultures 72 hours after medium was changed, and then centrifuged and stored at -80°C until used.

### Immunocytochemistry

Established CAFs were seeded on chamber slides and cultured for 48 hours. Cells were then fixed in 4% paraformaldehyde and permeabilized in 0.2% Triton X-100 at room temperature (RT). Cells were incubated with 10% donkey serum in phosphate-buffered saline (PBS) containing 0.2% Trion X-100 at RT for 30 min to block the unspecific binding of antibodies. After the blocking solution had been washed out, cells were incubated with primary antibodies, FAP (1:100; Abcam, Cambridge, UK), or α-SMA (1 : 100; Abcam) at RT for 1 hour. After being incubated at RT for 1 hour with a secondary antibody (Donkey Anti-Rabbit immunoglobulin G (IgG) H&L FITC; 1:1000; Abcam), the slides were mounted with fluoroshield mounting medium containing DAPI (Abcam), and sealed with nail polish. Slides were stored at 4°C in a dark box and observed under a fluorescent microscope.

### CD14-positive cell isolation and culture

Peripheral blood mononuclear cells (PBMCs) were prepared from healthy donor blood by density gradient centrifugation on Ficoll-Paque PLUS (GE Healthcare, Pittsburgh, PA, USA), followed by monocyte isolation using CD14 MicroBeads with an MS column (Miltenyi Biotec, Cologne, Germany). CD14-negative cells were stored at -80°C until used. One million isolated CD14-positive cells were plated onto a 6-well plate in 4.0 mL of culture supernatant from CAFs, which was half-diluted with RPMI supplemented with 10% FCS, 100 units/ml penicillin, and 100 μg/ml streptomycin (henceforth referred to as “conditioned RPMI”), or in 4.0 mL of DMEM supplemented with 10% FCS, 100 units/ml penicillin, and 100 μg/ml streptomycin (henceforth referred to as “conditioned DMEM”), which was also half-diluted with conditioned RPMI. After 48 hours of cultivation, CD14-positive cells co-cultured with the culture supernatants from CAFs (CAF-educated cells) and CD14-positive cells co-cultured with conditioned DMEM (control cells) were harvested by gently scraping the bottom of the wells, then examined by flow cytometry and real-time quantitative RT-PCR according to the methods described below.

### Flow cytometry

The non-specific binding of antibodies to Fc receptors on the harvested cells was blocked using BD Fc Block™ (BD Bioscience, San Jose, CA, USA) in accordance with the manufacturer's instructions, and cells were then stained with the following mouse anti-human antibodies in order to examine the expression of myeloid cell markers, HLA molecules, or co-regulatory molecules: HLA-DR, CD14, CD68, CD163, CD200R, CD206, CD80, CD86, HLA-G, B7H1/PD-L1, B7DC/PD-L2, or B7H3. All antibodies were directly conjugated to phycoerythrin (PE) or allophycocyanin (APC). Respective IgG isotype-matched controls were used as negative controls. Staining was performed, protected from light, at 4°C for 30 min in PBS with 1% heat-inactivated fetal calf serum (FCS; Life Technologies, Carlsbad, CA, USA) and 0.1% sodium azide (Sigma-Aldrich, St. Louis, MO, USA). Antibodies were purchased from BD Bioscience, BioLegend (San Diego, CA, USA) and eBioscience (San Diego, CA, USA). A Cytofix/Cytoperm™ Kit (BD Bioscience) was used to perform intracellular staining for CD68. After washing, samples were immediately analyzed by flow cytometry using an Attune^®^ Acoustic Focusing Cytometer (Life Technologies). Acquired data were analyzed using FlowJo software (TreeStar, Ashland, OR, USA).

### Real-time qRT-PCR

Total RNA was extracted from harvested cells using an RNeasy mini kit (Qiagen, Valencia, CA, USA). Quantitative RT-PCR was performed in triplicate using a Power SYBR Green RNA-to-CT 1-Step Kit on StepOne (Applied Biosystems, Foster City, CA, USA). A melting curve was recorded at the end of every run to assess the product specificity. Glyceraldehyde-3-phosphate dehydrogenase (*GAPDH*) was used as an internal control gene. Relative expression levels were measured by the 2-ΔΔCt method, in which Ct represented the threshold cycle. The PCR primers used in this study are shown in Supplementary Table 1.

### Carboxyfluorescein succinimidyl ester (CFSE)-based suppression assay

In order to evaluate the suppressive function of CAF-educated cells on T cells, CFSE-labeled CD14-negative PBMCs were cultured with CAF-educated cells or control cells. Isolated CD14-positive cells were plated at 5×10^4^, 2.5×10^4^, or 1.25×10^4^ cells per well on a 96-well flat bottom plate in 200 μL of culture supernatant from CAFs or conditioned DMEM, which was half-diluted with conditioned RPMI. After 48 hours of cultivation, cryopreserved autologous CD14-negative cells were thawed and washed, then incubated with 0.5 μM CFSE (Molecular Probe/Invitrogen, Life Technologies) at 37°C for 10 min, and quenched with ice-cold conditioned RPMI. After standing at RT for 5 min in the dark, cells were centrifuged, washed 3 more times, and then added at 10×10^4^ cells per well to a 96-well plate, as described above, after the wells had been washed with PBS. After 96 hours of co-cultivation with an anti-CD3/anti-CD28 stimulus (Treg Suppression Inspector human; Miltenyi Biotec), floating CFSE-labeled cells were harvested, then stained with APC-CD3 (BD Bioscience), APC-CD4 (BD Bioscience), or APC-CD8 (BD Bioscience), followed by staining with 7-amino-actinomycin D (7-AAD; BD Bioscience) 10 min before analysis. The proliferation of T cells was analyzed by the dilution of CFSE staining intensity using flow cytometry. Viable T cells were gated based on positive CD3 staining and negative 7-AAD staining.

Blocking assays for the suppressive function of CAF-educated cells were conducted at 1:4 CAF-educated cells:CD14-negative cells. Anti-IL-10 neutralizing mAb (10 μg/ml; eBioscience), anti-TGF-β neutralizing mAb (10 μg/ml; R&D Systems), Nɷ-hydroxy-nor-L-arginine (1μM; Noha, Cayman Chemical, MI, USA), or L-arginine (0.5mg/ml, Sigma-Aldrich) was added during 96 hours of co-cultivation, respectively.

### Patients and samples

Seventy-three resected OSCC samples that only consisted of primary tongue cancer were analyzed using IHC in the present study. All samples were obtained from patients who underwent surgery without preoperative neoadjuvant chemotherapy or radiation at Gunma University Hospital between November 2000 and January 2012. All samples were classified according to the WHO classification by a pathologist who was blind to the clinical findings, and were diagnosed as squamous cell carcinoma. The pathological TNM classification was established using the International System for Staging adopted by the American Joint Committee on Cancer and the Union Internationale Centre le Cancer (UICC). Clinicopathological variables, including age, the differentiation of tumors, lymphatic/vascular invasion, lymph node involvement, T factor, TNM stage, Ki-67 staining, and p53 staining, as well as OS and PFS, were also evaluated. This study was approved by the Institutional Review Board of Gunma University (No. 12-12), and was performed in line with the Declaration of Helsinki of 1996. All patients provided informed written consent.

### Immunohistochemistry

Surgical specimens were fixed in 10% formaldehyde and routinely processed for paraffin embedding. Serial histological sections (thickness of 5 μm) were deparaffinized in xylene and hydrated in descending dilutions of ethanol. Antigen retrieval was achieved by autoclaving at 121°C for 20 min in citrate buffer (pH 6.0) for α-SMA, CD163, Ki-67, and p53, or by the application of Proteinase K (Dako, Glostrup, Denmark) at RT for 5 min for CD68 staining. Endogenous peroxidase was blocked by 3% H_2_O_2_, and sections were then covered with 1% BSA/5% normal horse serum at RT for 30 min. The slides were incubated at 4°C overnight with monoclonal mouse anti-human α-SMA mAb (clone 1A4; 1:20; R&D Systems, Minneapolis, MN, USA), monoclonal mouse anti-human CD163 mAb (clone 10D6; 1:200; Leica Biosystems, Nussloch, Germany), monoclonal mouse anti-human CD68 mAb (clone PG-M1; ready-to-use; Dako), monoclonal mouse anti-human Ki-67 mAb (clone Ki-67P; 1:300; Dianova, Hamburg, Germany), and monoclonal mouse anti-human p53 mAb (clone DO-7; 1:50; Dako), followed by Labeled Polymer-HRP anti-mouse/rabbit (Dako) at RT for 45 min. Reaction products were detected by 3.3’-diaminobenzidine (DAB, Dako), and then counterstained using Mayer's hematoxylin (Wako Pure Chemical Industries, Ltd., Osaka, Japan). After dehydration by ascending dilutions of ethanol, the slides were mounted with the non-aqueous mounting medium DPX (Merck, Darmstadt, Germany).

### Evaluation of IHC samples

Slides were evaluated by two independent investigators (H.T. and K.S.) in a blinded manner using a light microscope, Axioscope (Carl Zeiss microscopy GmbH, Jena, Germany). The acquisition of images was performed using AxioVision LE (Carl Zeiss). Based on the method of previous studies [29, 34], the proportion of α-SMA positive fibroblasts was classified into four grades: negative (0), no CAFs; scanty (1), a small number of scattered CAFs; focal (2), concentrated CAFs with an irregular and non-continuous focus; and abundant (3), concentrated CAFs with an extensive and continuous focus. Each specimen was scored at the highest grade throughout the entire OSCC invasive stroma at ×100 magnification. More than four areas of a representative field adjacent to cancer cells were counted at ×200 magnification for CD68+ and CD163+ macrophages, and the average was calculated. According to a previous study, a highly cellular area of stained sections was evaluated for Ki-67 [40]. Approximately 1000 nuclei were counted on each slide, and proliferative activity was assessed as the percentage of Ki-67-stained nuclei (Ki-67 labeling index) in the sample. Based on previous studies [41], the expression of p53 in more than 10% of tumor cells was defined as positive expression.

### Statistical analysis

Data were analyzed using the Statistical Package for Social Science version 22.0 (SPSS, IBM, Armonk, NY, USA) and GraphPad Prism version 6.0 for Windows (GraphPad Software, San Diego, CA, USA). The Mann-Whitney U test, Kruskal-Wallis test, chi-squared test for independence, and Fisher's exact test were used to examine differences in continuous and categorical variables. Two-sided *P* values < 0.05 were considered to be significant. Univariate/multivariate regression analyses were performed using Cox's proportional hazards model. Survival curves were analyzed by Kaplan-Meier method and compared using the Log-rank test.

## SUPPLEMENTARY MATERIALS TABLE



## Abbreviations

7-AAD: 7-amino-actinomycin D
α-SMA: α-smooth muscle actin
APC: Allophycocyanin
CAF: Cancer-associated fibroblast
CFSE: Carboxyfluorescein succinimidyl ester
ECM: Extracellular matrix
FAP: Fibroblast activation protein
FCS: Fetal calf serum
GAPDH: Glyceraldehyde-3-phosphate dehydrogenase
HLA: Human leukocyte antigen
IgG: Immunoglobulin G
IHC: Immunohistochemistry
IL: Interleukin
Noha: Nɷ-hydroxy-nor-L-arginine
OS: Overall survival
OSCC: Oral squamous cell carcinoma
PBMC: Peripheral blood mononuclear cell
PE: Phycoerythrin
PFS: Progression-free survival
TAM: Tumor-associated macrophage
TGF-β: Transforming growth factor beta
TME: Tumor microenvironment
TNM: Tumor-node-metastasis
