# Supplementary material for: Cancer-associated fibroblasts promote an immunosuppressive microenvironment through the induction and accumulation of protumoral macrophages
Source: Oncotarget. 2016 Dec 30;8(5):8633–47. doi: 10.18632/oncotarget.14374 (PMC5352428; doi:10.18632/oncotarget.14374)
Supplement: Supplementary file 1 [file oncotarget-08-8633-s001.pdf]

Cancer-associated fibroblasts promote an immunosuppressive microenvironment through the induction and accumulation of protumoral macrophages

SUPPLEMENTARY TABLE

Supplementary Table S1: List of primers used for quantitative RT-PCR in this study

Gene	Forward primer	Reverse primer
ARG1	5'-AAAGGCTGGTCTGCTTGAGAA-3'	5'-GTCATTAGGGATGTCAGCAAAGG-3'
IL10	5'-GAGATGCCTTCAGCAGAGTGAAGA-3'	5'-AGGCTTGGCAACCCAGGTAAC-3'
TGFB1	5'-AGCGACTCGCCAGAGTGGTTA-3'	5'-GCAGTGTGTTATCCCTGCTGTCA-3'
VEGFA	5'-ACTTCCCCAAATCACTGTGG-3'	5'-GTCACTCACTTTGCCCCTGT-3'
TNF	5'-TGCTTGTTCCTCAGCCTCTT-3'	5'-CAGAGGGCTGATTAGAGAGAGGT-3'
IL1B	5'-TCGCCAGTGAAATGATGGCTTA-3'	5'-GTCCATGGCCACAACAACTGA-3'
GAPDH	5'-GCACCGTCAAGGCTGAGAAC-3'	5'-ATGGTGGTGAAGACGCCAGT-3'
